# Hydrogenation of the Exocyclic Olefinic Bond at C-16/C-17 Position of *ent*-Kaurane Diterpene Glycosides of *Stevia rebaudiana* Using Various Catalysts

**DOI:** 10.3390/ijms140815669

**Published:** 2013-07-26

**Authors:** Venkata Sai Prakash Chaturvedula, Indra Prakash

**Affiliations:** Organic Chemistry Department, Global Research and Development, The Coca-Cola Company, One Coca-Cola Plaza, Atlanta, GA 30313, USA; E-Mail: vchaturvedula@coca-cola.com

**Keywords:** *ent*-Kaurane diterpene glycosides, *Stevia rebaudiana*, catalytic hydrogenation, Pt/C, Pd(OH)_2_, Rh/C, Raney Ni, PtO_2_, 5% Pd/BaCO_3_, structure characterization

## Abstract

Catalytic hydrogenation of the exocyclic double bond present between C16 and C17 carbons of the four *ent*-kaurane diterpene glycosides namely rebaudioside A, rebaudioside B, rebaudioside C, and rebaudioside D isolated from *Stevia rebaudiana* has been carried out using Pt/C, Pd(OH)_2_, Rh/C, Raney Ni, PtO_2_, and 5% Pd/BaCO_3_ to their corresponding dihydro derivatives with 17α and 17β methyl group isomers. Reactions were performed using the above-mentioned catalysts with the solvents methanol, water, and ethanol/water (8:2) under various conditions. Synthesis of reduced steviol glycosides was performed using straightforward chemistry and their structures were characterized on the basis of 1D and 2D NMR spectral data, including a comparison with reported spectral data.

## 1. Introduction

**Figure 1:**
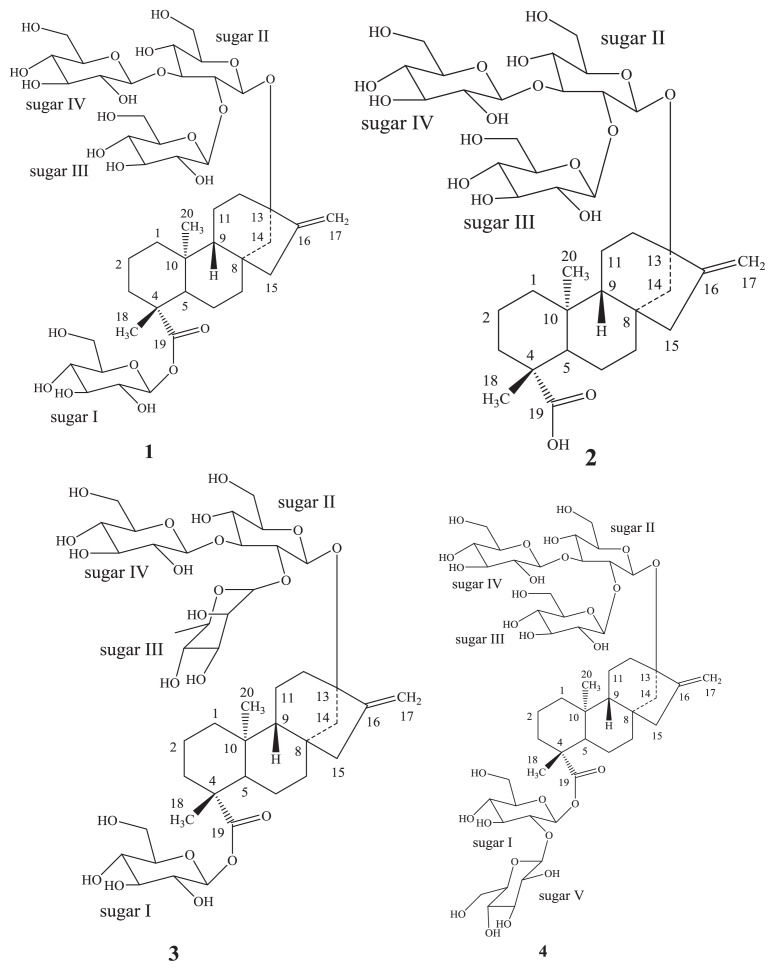
Structures of Rebaudioside A (**1**); Rebaudioside B (**2**); Rebaudioside C (**3**); and Rebaudioside D (**4**).

The major constituents isolated from the leaves of *Stevia rebaudiana* Bertoni (family: Asteraceae) are the potently sweet diterpenoid glycosides, stevioside, and rebaudioside A. These compounds are glycosides of the diterpene steviol, *ent*-13-hydroxykaur-16-en-19-oic acid, which are known as Stevia sweeteners [1] and are used to sweeten food products and beverages. Rebaudioside A (**1**) tastes about 200–300 times sweeter than sucrose; rebaudioside B (**2**) tastes about 150 times sweeter than sucrose; rebaudioside C (**3**) tastes about 20–30 times sweeter than sucrose, and rebaudioside D (**4**) tastes about 200–220 times sweeter than sucrose; all are non-caloric. Rebaudioside A (**1**) has a (2-*O*-β-d-glucopyranosyl-3-*O*-β-d-glucopyranosyl)-β-d-glucopyranosyl unit at the C-13 position with a β-d-glucopyranosyl moiety at the C-19 position of the aglycone steviol as an ester; rebaudioside B (**2**) has a (2-*O*-β-d-glucopyranosyl-3-*O*-β-d-glucopyranosyl)-β-d-glucopyranosyl unit at the C-13 position with a free carboxylic acid group at the C-19 position of the aglycone steviol; whereas rebaudioside C (**3**) has a (2-*O*-α-l-rhamnopyranosyl-3-*O*-β-d-glucopyranosyl)-β-d-glucopyranosyl unit at the C-13 position and a β-d-glucosyl moiety at the C-19 position of the aglycone steviol in the form of an ester; and rebaudioside D (**4**) has a (2-*O*-β-d-glucopyranosyl-3-*O*-β-d-glucopyranosyl)-β-d-glucopyranosyl unit at the C-13 position and a 2-*O*-β-d-glucopyranosyl-β-d-glucopyranosyl moiety at the C-19 position of the aglycone steviol as an ester (Figure 1).

As a part of our continuing research to discover natural sweeteners, we have reported several glycosides from the commercial extract of *S. rebaudiana* [2–6]. Apart from isolating novel compounds from *S. rebaudiana* and utilizing them as possible natural sweeteners or sweetness enhancers, we are also engaged in understanding the physicochemical profiles of steviol glycosides in various systems of interest and the structural characterization of their metabolites as well as their synthesis [7,8]. Recently, we have published the hydrogenation of the *ent*-kaurane diterpene glycosides namely rubusoside, stevioside, rebaudioside A, rebaudioside B, rebaudioside C, and rebaudioside D isolated from *S. rebaudiana* using Pd(OH)_2_ and their sensory evaluation [9,10]. In this article, we present the synthesis of *ent*-kaurane diterpene glycosides that are prepared by the reduction of the C-16/C-17 exocyclic double bond of rebaudioside A, rebaudioside B, rebaudioside C, and rebaudioside D using Pt/C, Pd(OH)_2_, Rh/C, Raney Ni, PtO_2_, and 5% Pd/BaCO_3_ as catalysts to their corresponding dihydro derivatives with the solvents methanol, water, and ethanol/water (8:2) under various experimental conditions. Their structures were characterized on the basis of extensive NMR and MS spectroscopic data as well as comparison of spectral data reported from literature.

## 2. Results and Discussion

### 2.1. Chemistry

**Scheme 1:**
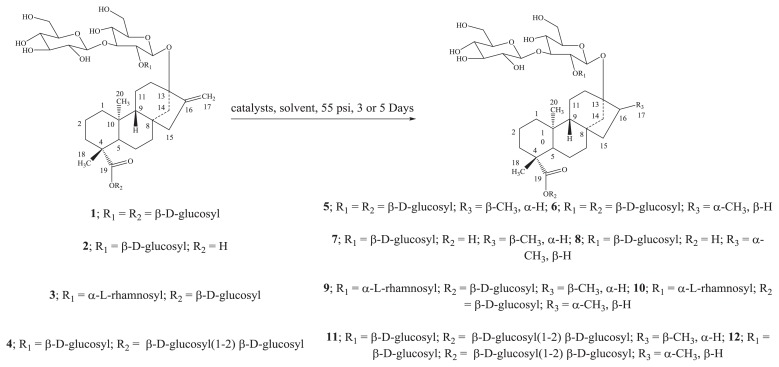
Hydrogenation of Rebaudioside A (**1**); Rebaudioside B (**2**); Rebaudioside C (**3**); and Rebaudioside D (**4**) using various catalysts and their reduced compounds.

Reduction of the four compounds rebaudioside A (**1**), rebaudioside B (**2**), rebaudioside C (**3**), and rebaudioside D (**4**), were performed using catalytic hydrogenation with Pt/C, Pd(OH)_2_, Rh/C, Raney Ni, PtO_2_, and 5% Pd/BaCO_3_ as catalysts in solvents of MeOH, H_2_O, and EtOH/H_2_O (8:2) at room temperature under 55 psi at 3 and 5 days furnished mixtures of dihydro derivatives of their corresponding isomers of 17α and 17β methyl groups (Scheme 1).

The conditions used for the catalytic hydrogenation of the four steviol glycosides rebaudioside A (**1**), rebaudioside B (**2**), rebaudioside C (**3**), and rebaudioside D (**4**) using Pt/C for the six methods along with the yields obtained for each reaction are given in Table 1.

From the above table, it has been observed that the reaction was completed between 69.0% and 83.0% yield after 3 days (72 h) with highest conversion to the reduced compounds using H_2_O as the solvent for the reactions. The conversion rate was similar using all three solvents after 5 days of catalytic reaction.

The conditions used for 5% Pd/BaCO_3_ as a catalyst towards the hydrogenation of the four steviol glycosides rebaudioside A (**1**), rebaudioside B (**2**), rebaudioside C (**3**), and rebaudioside D (**4**) and their methods along with the yields obtained for each reaction are given in Table 2.

From the above table, it was inferred that the reaction was completed between 64.0% and 78.0% after 3 days (72 h) with highest conversion to the reduced compounds with H_2_O as the reaction solvent using 10% Pd-BaCO_3_ as catalyst, whereas the conversion rate was in the same range using all three solvents MeOH, H_2_O, and EtOH-H_2_O (8:2) mixtures for 5 days of reaction.

The conditions used for the catalytic hydrogenation of the four steviol glycosides rebaudioside A (**1**), rebaudioside B (**2**), rebaudioside C (**3**), and rebaudioside D (**4**) using Rh/C for their six methods along with the yields obtained for each reaction are given in Table 3.

From Table 3, it was inferred that the hydrogenation reaction was completed between 62.0% and 81.0% after 3 days (72 h) with highest conversion to the reduced compounds of rebaudioside A (**1**), rebaudioside B (**2**), rebaudioside C (**3**), and rebaudioside D (**4**) using H_2_O as the solvent for the reactions with Rh/C as catalyst, whereas the conversion rate was similar using the two solvents MeOH, and H_2_O between 99.0% and 99.8% conversion, and around 97.0% in case of EtOH/H_2_O (8:2) mixture after 5 days reactions.

The experimental conditions used for the catalytic hydrogenation with Raney Ni on the four steviol glycosides rebaudioside A (**1**), rebaudioside B (**2**), rebaudioside C (**3**), and rebaudioside D (**4**) and the methods along with their yields for each reaction are given in Table 4.

From Table 4, it was identified that the reaction was completed between 57.0% and 81.2% after 3 days (72 h) with the highest conversion to the reduced compounds of rebaudioside A (**1**), rebaudioside B (**2**), rebaudioside C (**3**), and rebaudioside D (**4**) using H_2_O as the solvent for the reactions with Rh/C as catalyst. The conversion rate was similar using the two solvents MeOH, and H_2_O between 99.0% and 99.8% conversion, and around 98% in case of EtOH-H_2_O (8:2) mixture after 5 days reactions.

The conditions used for the catalytic hydrogenation of the four steviol glycosides rebaudioside A (**1**), rebaudioside B (**2**), rebaudioside C (**3**), and rebaudioside D (**4**) using PtO_2_ for their reaction methods along with the yields obtained are given in Table 5.

From the above table it was observed that the reaction was completed between 55.2% and 81.0% after 3 days (72 h) with highest conversion to the reduced compounds using H_2_O as the solvent for the reactions with PtO_2_ as catalyst. The conversion rate was almost same using all three solvents MeOH, H_2_O, and EtOH/H_2_O (8:2) mixtures for 5 days reaction.

The conditions used for the hydrogenation using Pd(OH)_2_ as catalyst on the four steviol glycosides rebaudioside A (**1**), rebaudioside B (**2**), rebaudioside C (**3**), and rebaudioside D (**4**) along with their methods and yields obtained for each reaction are given in Table 6.

From Table 6, it was concluded that the reaction was completed between 62.0% and 84.4% after 3 days (72 h) with highest conversion to the reduced compounds using H_2_O as the solvent for the reactions with PtO_2_ as catalyst, whereas the conversion rate was similar using all three solvents MeOH, H_2_O, and EtOH-H_2_O (8:2) mixtures after 5 days of reaction.

Further trails to separate the mixtures of the dihydro products of the hydrogenation of the four compounds: rebaudioside A (**1**), rebaudioside B (**2**), rebaudioside C (**3**), and rebaudioside D (**4**) using various separation techniques of preparative TLC and reversed phase HPLC failed. Hence we have reported these compounds as is.

### 2.2. Spectroscopy

The structural characterization of **5**–**12** were performed on the basis of one dimensional (^1^H, ^13^C), two-dimensional (^1^H–^1^H COSY, ^1^H–^13^C HMQC, ^1^H–^13^C HMBC) NMR and in comparison with the reported spectral data [9,10]. The stereochemistry at the C-16 position was identified in comparison with their corresponding aglycone derivative literature values [9–13] as well as chemical studies. The ^1^H and ^13^C NMR values for all the protons and carbons in **5**–**12** were assigned on the basis of COSY, HMQC and HMBC correlations. Further it was found that the ratio of 17α/17β reduced compounds were observed at an approximately 60:40 ratio based on NMR spectral data of the mixture after 3 or 5 days reduction with catalytic hydrogenation of rebaudioside A (**1**), rebaudioside B (**2**), rebaudioside C (**3**), and rebaudioside D (**4**) using Pt/C, Pd(OH)_2_, Rh/C, Raney Ni, PtO_2_, and 5% Pd/BaCO_3_ indicates that the ratio is devoid of catalysts. The ^1^H NMR data for the key protons in **5**–**12** are given in Table 7, whereas the complete assignments of their carbon values are given in Table 8.

## 3. Experimental Section

### 3.1. General

Samples of the synthesized steviol glycosides **5**–**12** are available from the authors. Melting points were measured using a SRS Optimelt MPA 100 instrument and are uncorrected. IR spectral data was acquired using a Perkin Elmer 400 Fourier Transform Infrared Spectrometer (Perkin Elmer, Waltham, MA, USA) with Universal attenuated total reflectance (UATR) polarization accessory. NMR spectra were acquired a Varian Unity Plus 600 MHz instrument in C_5_D_5_N using standard pulse sequences. Chemical shifts are given in δ (ppm), and coupling constants are reported in Hz. HRMS and MS/MS data were generated with a Waters Premier Quadrupole Time-of-Flight mass spectrometer (Waters Corporation, Milford, MA, USA) equipped with an electrospray ionization source operated in the positive-ion mode and ThermoFisher Discovery OrbiTrap in the positive mode of electrospray. Samples were diluted with water/acetonitrile (1:1) containing 0.1% formic acid and introduced via infusion using the onboard syringe pump. The mixed solvents were made up on a volume/volume basis.

### 3.2. Isolation of Reduced Steviol Glycosides **5–12**

#### 3.2.1. General Procedure for the Catalytic Hydrogenation of Steviol Glycosides **1**–**4** with Pd(OH)_2_

To a solution of each steviol glycoside **1**–**4** (2 g) dissolved in 100 mL of solvent was added Pd(OH)_2_ (50 mg). The mixture was hydrogenated at ambient temperature under 55 psi. At the end of hydrogenation (3 or 5 days), the reaction mixture was filtered through celite and concentrated under vacuum to afford a clear white product. The product, triturated in acetone and filtered and dried under vacuum at 50 °C for 2 days furnished the corresponding dihydroderivatives of **1**–**4**.

#### 3.2.2. General Procedure for the Catalytic Hydrogenation of Steviol Glycosides **1**–**4** with Pt/Charcoal

To a solution of each steviol glycoside **1**–**4** (2 g) dissolved in 100 mL of solvent was added Pt/C (100 mg). The mixture was hydrogenated at ambient temperature under 55 psi. At the end of hydrogenation (3 or 5 days), the reaction mixture was filtered in nitrogen atmosphere under vacuum (failing to do this would result in fire of the reaction mixture) and concentrated under vacuum to afford a clear white product. The product was triturated in acetone, filtered and dried under vacuum at 50 °C for 2 days which yielded their corresponding dihydroderivatives.

#### 3.2.3. General Procedure for the Catalytic Hydrogenation of Steviol Glycosides **1**–**4** with Rh/Charcoal

To a solution of each steviol glycoside **1**–**4** (2 g) dissolved in 100 mL of solvent was added Rh/C (100 mg). The mixture was hydrogenated at ambient temperature under 55 psi. At the end of hydrogenation (3 or 5 days), the reaction mixture was filtered through celite and concentrated under vacuum to afford a clear white product. The product was triturated in acetone/diethylether (1:1) and filtered. The product obtained was dried under vacuum at 50 °C for 2 days which yielded their corresponding dihydroderivatives.

#### 3.2.4. General Procedure for the Catalytic Hydrogenation of Steviol Glycosides **1**–**4** with Raney Ni

To a solution of each steviol glycoside **1**–**4** (2 g) dissolved in 100 mL of solvent was added Raney Ni (100 mg). The mixture was hydrogenated at ambient temperature under 55 psi. At the end of hydrogenation (3 or 5 days), the reaction mixture was filtered in nitrogen atmosphere under vacuum (failing to do this would result in fire of the reaction mixture) and concentrated under vacuum to afford a clear white product. The product was triturated with acetone/ethylmethylketone (1:1), filtered and dried under vacuum at 50 °C for 2 days yielding the corresponding dihydroderivatives of **1**–**4**.

#### 3.2.5. General Procedure for the Catalytic Hydrogenation of Steviol Glycosides **1**–**4** with 5% Pd/BaCO_3_

To a solution of each steviol glycoside **1**–**4** (2 g) dissolved in 100 mL of solvent was added 5% Pd/BaCO_3_ (100 mg). The mixture was hydrogenated at ambient temperature under 55 psi. At the end of hydrogenation (3 or 5 days), the reaction mixture was filtered through celite and concentrated under vacuum to afford a clear white product. The product was triturated in acetone/diethylether (1:1) and filtered and dried under vacuum at 50 °C for 2 days yielding the corresponding dihydroderivatives of **1**–**4**.

#### 3.2.6. General Procedure for the Catalytic Hydrogenation of Steviol Glycosides **1**–**4** with PtO_2_

To a solution of each steviol glycoside **1**–**4** (2 g) dissolved in 100 mL of solvent was added PtO_2_ (50 mg). The mixture was hydrogenated at ambient temperature under 55 psi. At the end of hydrogenation (3 or 5 days), the reaction mixture was filtered in nitrogen atmosphere under vacuum (failing to do this would result in fire of the reaction mixture) and concentrated under vacuum to afford a clear white product. The product was triturated in acetone, filtered and dried under vacuum at 50 °C for 2 days which yielded their corresponding dihydroderivatives.

The structural characterization of each isomeric mixture of (**5**/**6**), (**7**/**8**), (**9**/**10**), and (**11**/**12**) obtained by the catalytic hydrogenation of rebaudioside A (**1**), rebaudioside B (**2**), rebaudioside C (**3**), and rebaudioside D (**4**) using Pt/C, Pd(OH)_2_, Rh/C, Raney Ni, PtO_2_, and 5% Pd/BaCO_3_ has been confirmed by co-HPLC and comparative spectral data of the reported literature data [9,10].

#### 3.2.7. Dihydrorebaudiososide A1/Dihydrorebaudiososide A2 (**5**/**6**)

White powder; IR νmax: 3342, 2928, 2886, 1735, 1033, 887 cm^−1; 1^H NMR (600 MHz, C_5_D_5_N, δ ppm) and ^13^C NMR (150 MHz, C_5_D_5_N, δ ppm) spectroscopic data see Tables 7 and 8; HRMS (M + H)^+^*m*/*z* 969.4553 (calcd. for C_44_H_73_O_23_: 969.4543), (M + NH_4_)^+^*m*/*z* 986.4808 (calcd. for C_44_H_76_NO_23_: 986.4808) [9].

#### 3.2.8. Dihydrorebaudioside B1/Dihydrorebaudioside B2 (**7**/**8**)

White powder; IR ν_max_: 3345 cm^−1^, 2932 cm^−1^, 2880 cm^−1^, 1726 cm^−1^, 1036 cm^−1^, 894 cm^−1; 1^H-NMR and ^13^C-NMR spectroscopic data see Tables 7 and 8, respectively; HRMS (M + NH_4_)^+^*m*/*z* 824.4282 (calcd. for C_38_H_66_NO_18_: 824.4280), (M + Na)^+^*m*/*z* 829.3838 (calcd. for C_38_H_62_O_18_Na: 829.3834) [10].

#### 3.2.9. Dihydrorebaudioside C1/Dihydrorebaudioside C2 (**9**/**10**)

White powder; IR ν_max_: 3355 cm^−1^, 2930 cm^−1^, 2883 cm^−1^, 1724 cm^−1^, 1034 cm^−1^, 892 cm^−1; 1^H-NMR and ^13^C-NMR spectroscopic data see Tables 7 and 8, respectively; HRMS (M + NH_4_)^+^*m*/*z* 970.4864 (calcd. for C_44_H_76_NO_22_: 970.4859), (M + Na)^+^*m*/*z* 975.4418 (calcd. for C_44_H_72_NaO_22_: 975.4413) [10].

#### 3.2.10. Dihydrorebaudioside D1/Dihydrorebaudioside D2 (**11**/**12**)

White powder; IR ν_max_: 3348 cm^−1^, 2923 cm^−1^, 2882 cm^−1^, 1726 cm^−1^, 1033 cm^−1^, 880 cm^−1; 1^H-NMR and ^13^C-NMR spectroscopic data see Tables 7 and 8, respectively; HRMS (M + H)^+^*m*/*z* 1131.5074 (calcd. for C_50_H_83_O_28_: 1131.5071), (M + NH_4_)^+^*m*/*z* 1148.5342 (calcd. for C_50_H_86_NO_28_: 1148.5336) [10].

## 4. Conclusions

In conclusion, eight *ent*-kaurane diterpene glycosides **5**–**12** were synthesized by catalytic hydrogenation of the four natural products rebaudioside A (**1**), rebaudioside B (**2**), rebaudioside C (**3**), and rebaudioside D (**4**) using Pt/C, Pd(OH)_2_, Rh/C, Raney Ni, PtO_2_, and 5% Pd/BaCO_3_ to their corresponding dihydro derivatives with 17α and 17β methyl group isomers with solvents methanol, water, and ethanol/water (8:2) under various conditions. The structures of all synthesized compounds were characterized on the basis of NMR (1D and 2D) and mass spectral data, as well as in comparison with the data reported in the literature. This is the first report of the catalytic hydrogenation of rebaudioside A, rebaudioside B, rebaudioside C, and rebaudioside D under various experimental conditions of solvents and duration of reaction time.

## Figures and Tables

**Table 1:** Catalytic hydrogenation of Rebaudioside A (**1**); Rebaudioside B (**2**); Rebaudioside C (**3**); and Rebaudioside D (**4**) using Pt/C and yields of various methods.

S. No.	Solvent	Conditions	Duration	Yield
Method 1	MeOH	Temp.: 25 °C; Pressure: 55 psi	72 h	72.2%–77.5%
Method 2	MeOH	Temp.: 25 °C; Pressure: 55 psi	120 h	99.0%–99.5%
Method 3	H_2_O	Temp.: 25 °C; Pressure: 55 psi	72 h	78.0%–83.0%
Method 4	H_2_O	Temp.: 25 °C; Pressure: 55 psi	120 h	99.0%–99.8%
Method 5	EtOH/H_2_O (8:2)	Temp.: 25 °C; Pressure: 55 psi	72 h	69.0%–72.5%
Method 6	EtOH/H_2_O (8:2)	Temp.: 25 °C; Pressure: 55 psi	120 h	99.2%–99.5%

**Table 2:** Catalytic hydrogenation of Rebaudioside A (**1**), Rebaudioside B (**2**), Rebaudioside C (**3**), and Rebaudioside D (**4**) using 5% Pd/BaCO_3_ and yields of various methods.

S. No.	Solvent	Conditions	Duration	Yield
Method 1	MeOH	Temp.: 25 °C; Pressure: 55 psi	72 h	64.0%–69.5%
Method 2	MeOH	Temp.: 25 °C; Pressure: 55 psi	120 h	99.0%–99.5%
Method 3	H_2_O	Temp.: 25 °C; Pressure: 55 psi	72 h	72.2%–78.0%
Method 4	H_2_O	Temp.: 25 °C; Pressure: 55 psi	120 h	98.8%–99.5%
Method 5	EtOH/H_2_O (8:2)	Temp.: 25 °C; Pressure: 55 psi	72 h	66.2%–70.5%
Method 6	EtOH/H_2_O (8:2)	Temp.: 25 °C; Pressure: 55 psi	120 h	99.0%–99.5%

**Table 3:** Catalytic hydrogenation of Rebaudioside A (**1**); Rebaudioside B (**2**); Rebaudioside C (**3**); and Rebaudioside D (**4**) using Rh/C and yields of various methods.

S. No.	Solvent	Conditions	Duration	Yield
Method 1	MeOH	Temp.: 25 °C; Pressure: 55 psi	72 h	62.0%–67.5%
Method 2	MeOH	Temp.: 25 °C; Pressure: 55 psi	120 h	99.0%–99.5%
Method 3	H_2_O	Temp.: 25 °C; Pressure: 55 psi	72 h	78.2%–81.0%
Method 4	H_2_O	Temp.: 25 °C; Pressure: 55 psi	120 h	99.5%–99.8%
Method 5	EtOH/H_2_O (8:2)	Temp.: 25 °C; Pressure: 55 psi	72 h	68.4%–73.2%
Method 6	EtOH/H_2_O (8:2)	Temp.: 25 °C; Pressure: 55 psi	120 h	96.5%–97.2%

**Table 4:** Catalytic hydrogenation of Rebaudioside A (**1**); Rebaudioside B (**2**); Rebaudioside C (**3**); and Rebaudioside D (**4**) using Raney Ni and yields of various methods.

S. No.	Solvent	Conditions	Duration	Yield
Method 1	MeOH	Temp.: 25 °C; Pressure: 55 psi	72 h	57.0%–61.2%
Method 2	MeOH	Temp.: 25 °C; Pressure: 55 psi	120 h	99.0%–99.5%
Method 3	H_2_O	Temp.: 25 °C; Pressure: 55 psi	72 h	76.5%–81.2%
Method 4	H_2_O	Temp.: 25 °C; Pressure: 55 psi	120 h	99.5%–99.8%
Method 5	EtOH-H_2_O (8:2)	Temp.: 25 °C; Pressure: 55 psi	72 h	68.5%–73.5%
Method 6	EtOH-H_2_O (8:2)	Temp.: 25 °C; Pressure: 55 psi	120 h	97.5%–98.4%

**Table 5:** Catalytic hydrogenation of Rebaudioside A (**1**); Rebaudioside B (**2**); Rebaudioside C (**3**); and Rebaudioside D (**4**) using PtO_2_ and yields of various methods.

S. No.	Solvent	Conditions	Duration	Yield (17β/17α)
Method 1	MeOH	Temp.: 25 °C; Pressure: 55 psi	72 h	55.2%–59.5%
Method 2	MeOH	Temp.: 25 °C; Pressure: 55 psi	120 h	99.0%–99.7%
Method 3	H_2_O	Temp.: 25 °C; Pressure: 55 psi	72 h	78.3%–81.5%
Method 4	H_2_O	Temp.: 25 °C; Pressure: 55 psi	120 h	99.0%–99.8%
Method 5	EtOH/H_2_O (8:2)	Temp.: 25 °C; Pressure: 55 psi	72 h	67.5%–73.0%
Method 6	EtOH/H_2_O (8:2)	Temp.: 25 °C; Pressure: 55 psi	120 h	99.3%–99.7%

**Table 6:** Catalytic hydrogenation of Rebaudioside A (**1**); Rebaudioside B (**2**); Rebaudioside C (**3**); and Rebaudioside D (**4**) using Pd(OH)_2_ and yields of various methods.

S. No.	Solvent	Conditions	Duration	Yield (17β/17α)
Method 1	MeOH	Temp.: 25 °C; Pressure: 55 psi	72 h	62.0%–67.5%
Method 2	MeOH	Temp.: 25 °C; Pressure: 55 psi	120 h	99.0%–99.5%
Method 3	H_2_O	Temp.: 25 °C; Pressure: 55 psi	72 h	81.2%–84.4%
Method 4	H_2_O	Temp.: 25 °C; Pressure: 55 psi	120 h	99.3%–99.8%
Method 5	EtOH/H_2_O (8:2)	Temp.: 25 °C; Pressure: 55 psi	72 h	65.4%–73.5%
Method 6	EtOH/H_2_O (8:2)	Temp.: 25 °C; Pressure: 55 psi	120 h	99.4%–99.9%

**Table 7:** ^1^H NMR chemical shifts values for reduced compounds **5**–**12** recorded in pyridine-d5 (C_5_D_5_N) a–c.

Position	5	6	7	8	9	10	11	12
17	1.10 (d, 6.4, 1H)	1.16 (d, 6.4, 1H)	1.15 (d, 6.6, 1H)	1.32 (d, 6.4, 1H)	1.18 (d, 6.5, 1H)	1.37 (d, 6.4, 1H)	1.14 (d, 6.6, 1H)	1.16 (d, 6.4, 1H)
18	1.26 (s, 3H)	1.25 (s, 3H)	1.15 (s, 3H)	1.17 (s, 3H)	1.24 (s, 3H)	1.27 (s, 3H)	1.14 (s, 3H)	1.15 (s, 3H)
20	1.31 (s, 3H)	1.32 (s, 3H)	1.17 (s, 3H)	1.34 (s, 3H)	1.29 (s, 3H)	1.28 (s, 3H)	1.41 (s, 3H)	1.42 (s, 3H)
Sugar I-1′	6.16 (d, 6.8, 1H)	6.16 (d, 6.6, 1H)			6.13 (d, 6.8, 1H)	6.14 (d, 6.5, 1H)	6.86 (d, 6.4, 1H)	6.84 (d, 6.5, 1H)
Sugar II-1″	5.02 (d, 6.7, 1H)	5.01 (d, 6.7, 1H)	5.04 (d, 6.6, 1H)	5.01 (d, 6.4, 1H)	5.10 (d, 6.7, 1H)	5.07 (d, 6.4, 1H)	5.50 (d, 6.6, 1H)	5.53 (d, 6.4, 1H)
Sugar III-1‴	5.36 (d, 6.7, 1H)	5.34 (d, 6.4, 1H)	5.33 (d, 6.4, 1H)	5.34 (d, 6.3, 1H)	5.92 (d, 6.4, 1H)	5.75 (d, 6.8, 1H)	5.52 (d, 6.6, 1H)	5.56 (d, 6.6, 1H)
Sugar IV-1‴′	5.51 (d, 6.4, 1H)	5.42 (d, 6.8, 1H)	5.47 (d, 6.1, 1H)	5.51 (d, 6.4, 1H)	6.51 (d, 1.8, 1H)	6.84 (d, 1.6, 1H)	5.36 (d, 6.4, 1H)	5.41 (d, 6.6, 1H)
Sugar V-1‴″					1.64 (d, 6.1, 3H)	1.72 (d, 6.4, 3H)	6.31 (d, 6.4, 1H)	6.33 (d, 6.2, 1H)

a assignments made on the basis of COSY, HSQC and HMBC correlations;

b Chemical shift values are in δ (ppm);

c Coupling constants are in Hz.

**Table 8:** ^13^C NMR chemical shifts values for reduced compounds **5**–**12** recorded in pyridine-d5 (C_5_D_5_N) a–b.

Position	5	6	7	8	9	10	11	12
1	41.1	41.3	40.3	40.2	41.4	41.3	41.2	41.2
2	20.2	20.2	20.3	20.5	20.3	20.2	20.4	20.3
3	38.8	38.7	38.7	38.7	38.8	38.8	38.7	38.6
4	44.5	43.1	44.3	43.2	44.4	43.4	44.4	42.9
5	57.7	57.7	57.6	55.8	58.0	58.1	58.1	58.0
6	22.9	23.0	23.1	23.4	22.8	23.1	23.0	23.0
7	41.6	40.3	41.6	40.4	41.6	40.2	41.4	40.2
8	44.5	43.2	44.3	43.2	44.4	43.1	43.5	42.5
9	55.7	54.8	55.8	50.6	56.4	54.6	55.4	55.1
10	40.2	40.3	40.2	40.3	40.3	40.3	40.1	40.3
11	20.3	20.7	20.5	20.8	20.4	20.7	20.6	20.7
12	35.4	44.4	35.5	44.0	35.4	44.2	35.9	44.0
13	88.3	88.2	88.6	88.2	86.2	86.2	88.3	88.1
14	47.3	50.5	47.6	50.6	47.1	50.3	47.5	51.1
15	47.2	44.6	47.7	44.2	47.1	44.9	47.6	44.8
16	41.2	38.9	41.5	38.7	41.2	39.0	41.1	38.9
17	14.5	19.7	16.5	16.4	14.2	19.7	14.4	17.2
18	28.6	28.6	29.7	29.8	28.6	28.6	29.4	29.8
19	177.4	177.7	180.5	180.3	177.8	177.6	176.5	176.3
20	15.6	15.8	15.8	16.2	15.7	16.1	15.6	16.0

Sugar I								

1′	96.3	96.2			96.2	95.9	96.2	96.3
2′	75.8	75.4			75.4	75.3	81.6	81.2
3′	79.6	79.8			79.0	79.3	78.9	78.7
4′	71.6	71.4			71.2	71.4	71.6	71.4
5′	78.6	78.8			78.5	78.6	78.5	78.6
6′	63.1	63.2			62.5	62.5	63.1	63.2

Sugar II								

1″	99.1	98.8	98.7	98.9	98.4	96.8	94.2	94.3
2″	78.4	78.5	78.8	78.6	78.4	78.6	79.1	79.2
3″	86.5	85.6	85.6	86.6	87.3	86.2	86.1	86.7
4″	71.9	72.1	72.1	72.2	70.6	70.6	71.2	71.1
5″	77.3	77.0	77.0	77.0	75.6	75.4	77.2	77.1
6″	62.7	62.9	62.9	62.8	62.7	62.7	62.8	62.9

Sugar III								

1‴	105.2	105.5	105.5	105.0	103.2	102.2	105.2	104.9
2‴	74.6	74.7	74.6	74.6	71.9	71.6	75.9	75.9
3‴	77.5	77.9	77.8	77.8	72.9	72.9	78.7	78.6
4‴	72.1	72.0	72.1	72.3	73.0	73.2	72.4	72.2
5‴	78.9	79.1	79.0	79.3	70.3	70.2	79.0	79.3
6‴	62.6	62.5	62.9	62.9	19.6	19.3	62.6	62.8

Sugar IV								

1‴′	105.4	105.9	105.6	105.5	105.2	104.8	105.2	105.7
2‴′	74.3	74.6	75.6	75.7	74.8	74.7	74.3	74.4
3‴′	79.9	79.9	81.8	81.2	79.8	79.9	79.8	79.7
4‴′	72.2	72.3	72.0	72.1	72.4	72.1	72.2	72.3
5‴′	79.8	79.7	79.1	79.2	79.0	78.7	79.6	79.8
6‴′	63.2	63.0	63.3	63.3	63.4	63.1	63.2	63.3

Sugar V								

1‴″							106.1	105.6
2‴″							76.8	76.8
3‴″							78.9	78.9
4‴″							72.3	72.2
5‴″							81.8	81.3
6‴″							63.5	63.5

a assignments made on the basis of COSY, HMQC and HMBC correlations;

b Chemical shift value are in δ (ppm).

## References

[1] Brandle J.E., Starrratt A.N., Gijen M. (1998). *Stevia rebaudiana*: Its agricultural, biological and chemical properties. Can. J. Plant Sci.

[2] Chaturvedula V.S.P., Rhea J., Milanowski D., Mocek U., Prakash I. (2011). Two minor diterpene glycosides from the leaves of *Stevia rebaudiana*. Nat. Prod. Commun.

[3] Chaturvedula V.S.P., Mani U., Prakash I. (2011). Diterpene glycosides from *Stevia rebaudiana*. Molecules.

[4] Chaturvedula V.S.P., Prakash I. (2011). A new diterpenoid glycoside from *Stevia rebaudiana*. Molecules.

[5] Chaturvedula V.S.P., Clos J.F., Rhea J., Milanowski D., Mocek U., DuBois G.E., Prakash I. (2011). Minor diterpenoid glycosides from the leaves of *Stevia rebaudiana*. Phytochem. Lett.

[6] Chaturvedula V.S.P., Mani U., Prakash I. (2011). Structures of the novel α-glucosyl linked diterpene glycosides from *Stevia rebaudiana*. Carbohydr. Res.

[7] Chaturvedula V.S.P., Klucik J., Mani U., Prakash I. (2011). Synthesis of *ent*-kaurane diterpene glycosides. Molecules.

[8] Prakash I., Clos J.F., Chaturvedula V.S.P. (2012). Stability of rebaudioside A under acidic conditions and its degradation products. Food Res. Intl.

[9] Chaturvedula V.S.P., Campbell M., Miguel R.I.S., Prakash I. (2012). Synthesis and sensory evaluation of *ent*-kaurane diterpene glycosides. Molecules.

[10] Chaturvedula V.S.P., Campbell M., Prakash I. (2012). Catalytic hydrogenation of the sweet principles of *Stevia rebaudiana*, rebaudioside B, rebaudioside C, and rebaudioside D and sensory evaluation of their reduced derivatives. Int. J. Mol. Sci.

[11] Kasai R., Kaneda N., Tanaka O., Yamasaki K., Sakamoto I., Morimoto K., Okada S., Kitahata S., Furukawa H. (1981). Sweet diterpene glycosides of leaves of *Stevia rebaudiana* Bertoni: Synthesis and structure-sweetness relation of rebaudiosides A, D, and E and their related glycosides. Nippon Kagaku Kaishi.

[12] Nanayakkara N.P.D., Klocke J.A., Compadre C.M., Hussain R.A., Pezzuto J.M., Kinghorn A.D. (1987). Characteriztaion and feeding deterrent effects on the aphid, *Schizaphis graminum*, of some derivatives of the sweet compounds, stevioside and rebaudioside A. J. Nat. Prod.

[13] Pezzuto J.M., Compadre C.M., Swanson S.M., Nanayakkara N.P.D., Kinghorn A.D. (1985). Metabolically activated steviol, the aglycone of stevioside, is mutagenic. Proc. Natl. Acad. Sci. USA.

